# Safety, Pharmacokinetics, and Pharmacodynamics of Etavopivat (FT‐4202), an Allosteric Activator of Pyruvate Kinase‐R, in Healthy Adults: A Randomized, Placebo‐Controlled, Double‐Blind, First‐in‐Human Phase 1 Trial

**DOI:** 10.1002/cpdd.1058

**Published:** 2022-01-12

**Authors:** Sanjeev Forsyth, Patricia Schroeder, James Geib, Leela Vrishabhendra, Diamantis G. Konstantinidis, Kari LaSalvia, Maria D. Ribadeneira, Eric Wu, Patrick Kelly, Theodosia A. Kalfa

**Affiliations:** ^1^ Forma Therapeutics, Inc. Watertown Massachusetts USA; ^2^ Medpace Clinical Pharmacology Unit Cincinnati Ohio USA; ^3^ Cincinnati Children's Hospital Medical Center Cincinnati Ohio USA; ^4^ Department of Pediatrics University of Cincinnati College of Medicine Cincinnati Ohio USA

**Keywords:** clinical trial, etavopivat, first‐in‐human study, pharmacodynamics, pharmacokinetics, safety, sickle cell disease

## Abstract

Etavopivat (FT‐4202) is an orally administered, small‐molecule allosteric activator of erythrocyte pyruvate kinase‐R (PKR) in clinical development for the treatment of sickle cell disease and other hemoglobin disorders. This randomized, placebo‐controlled, double‐blind, first‐in‐human combination single‐ascending dose and multiple‐ascending dose phase 1 trial (NCT03815695) evaluated the safety and pharmacokinetics/pharmacodynamics of etavopivat in 90 healthy adult subjects. In 4 single‐ascending dose cohorts, 8 participants were randomized 3:1 to a single oral dose of either etavopivat (n = 6) or placebo (n = 2). In four 14‐day multiple‐ascending dose cohorts, 12 participants were randomized 3:1 to 14 days of etavopivat (n = 9) or placebo (n = 3). In these studies, most treatment‐emergent adverse events were of mild severity (grade 1) and none led to study discontinuation. Etavopivat exhibited a linear and time‐independent pharmacokinetic profile (at doses ≤400 mg) and elicited the expected pharmacodynamic effects of PKR activation (decreased 2,3‐diphosphoglycerate and increased adenosine triphosphate) and evidence of improved hemoglobin‐oxygen affinity. In addition, pharmacodynamic responses were durable with effects continuing for 48 to 72 hours after the last dose, thereby supporting once‐daily dosing. Food appeared to have no clinically meaningful effects on etavopivat exposure, thus facilitating administration with or without food. In conclusion, the evaluation of etavopivat in healthy subjects demonstrated proof of mechanism (PKR activation) without significant adverse events. This study also allowed for the selection of dose levels, projected to have an acceptable safety profile and provide therapeutic benefit, for evaluation in future trials in patients with sickle cell disease.

Sickle cell anemia (SCA) is a red blood cell (RBC) disorder characterized by chronic hemolytic anemia and painful vaso‐occlusive crises. It represents the most common inherited hemolytic anemia, affecting ≈100 000 patients in the United States^1^ as well as millions of people across the globe, and this number is expected to continue to rise.^2^, ^3^, ^4^ SCA results from homozygous inheritance of a mutated form of β globin (β^S^), participating in a hemoglobin tetramer known as hemoglobin S (HbS). Other types of sickle cell disease (SCD) are associated with inheritance of a β^S^ gene in trans to another β globin mutation, leading, for example, to sickle β thalassemia (Sβ^o^ or Sβ^+^) or to HbSC disease.

The origin of the pathobiology of SCD is polymerization of HbS in deoxygenated RBCs, leading to rigid, sickle‐shaped RBCs, vaso‐occlusion, and hemolysis. In patients with SCD, adhesion of erythrocytes in postcapillary venules can slow or even arrest blood flow, leading to further deoxygenation in the capillaries, and thus formation of sickle RBCs. Mechanical obstruction by both rigid, sickle‐shaped RBCs and cell adhesion contributes to acute, painful vaso‐occlusive crises, resulting in tissue ischemia, hypoxia/reperfusion tissue injury and inflammation, infarction, and chronic and insidious long‐term organ damage.^5^ Circulating HbS RBCs are mechanically fragile. Increased metabolic demand associated with increased oxidation, dehydration, and phosphorylation of the cytoskeleton decreases their deformability and makes them increasingly susceptible to hemolysis, leading to chronic anemia.^6^, ^7^, ^8^ These sequelae contribute to the significant morbidity and increased mortality associated with SCD.^9^, ^10^

Treatment options for patients with SCD are currently limited, and additional therapies with the potential to target the underlying pathophysiology of SCD and forestall cumulative organ damage are needed. Recently, attention has been focused on pyruvate kinase‐R (PKR) as a potential therapeutic target. There are four isozymes of pyruvate kinase in human tissues; PKR is the isoform specific to RBCs, and a key enzyme in the glycolysis pathway.^11^ Activation of PKR inhibits HbS deoxygenation and RBC sickling by decreasing 2,3‐diphosphoglycerate (2,3‐DPG) and physiologically increasing the HbS oxygen affinity.^12^ Furthermore, PKR activation increases adenosine triphosphate (ATP), which has been shown to support RBC membrane integrity and stress resilience overall,^13^ thus potentially decreasing hemolysis. ATP also supports the reduction of reactive oxygen species, which can damage RBCs and impair their functionality, and it has been shown to reduce vascular adhesion associated with RBC membrane injury in a murine model.^14^ The multimodal effects of PKR activation, including the combination of antisickling effects, decreased hemolysis, and improved RBC membrane fitness, may potentially reduce the incidence of vaso‐occlusive crises and, in parallel, may potentially ameliorate chronic anemia in SCD.

Etavopivat (FT‐4202) is an investigational, orally bioavailable, small‐molecule allosteric activator of PKR. The chemical structure is shown in Figure S1. In SCA models, etavopivat treatment reduced RBC sickling and deformability, leading to an increase in RBC survival of nearly 30%.^15^ In RBCs from previously untreated patients with homozygous HbS, etavopivat increased the oxygen affinity of hemoglobin, with a mean decrease of 2.33 mm Hg in the partial pressure of oxygen at which Hb‐oxygen saturation of 50% is achieved (P_50_).^16^

In vitro, no safety signals for etavopivat were identified by the human ether‐à‐go‐go–related gene patch clamp assay (data on file at Forma). Mean (±SD) percent inhibition ranged from 1.4±1.2% for etavopivat 10 μM to 37±1.8% for 300 μM, and the half maximal inhibitory concentration was estimated to be >300 μM (data on file at Forma), relative to the observed maximum in vivo plasma concentrations of unbound drug following a single dose of 1000 mg (supratherapeutic dose) of 1.2 μM. In vitro cytochrome P450 (CYP) phenotyping studies in which etavopivat was incubated with 10 recombinant human CYP enzymes showed that etavopivat is metabolized via CYP2C9, CYP3A4, and CYP3A5, with intrinsic clearance values of 0.161, 1.35, and 0.190 μl/min/pmol of CYP, respectively, and half‐life (t_1/2_) values of 86.2, 10.3, and 73.3 minutes (data on file at Forma). In human liver microsomes incubated with 2 μM etavopivat and specific CYP inhibitors for 0, 15, 30, 45, and 60 minutes, the remaining percentage of etavopivat was >80% both with and without specific inhibitors for CYP1A2, CYP2B6, CYP2C8, CYP2C9, CYP2C19, CYP2D6, and CYP3A at all time points, indicating that etavopivat was relatively stable. In vitro drug interaction assessments conducted in human hepatocytes confirmed that etavopivat does not induce CYP3A4, CYP2B6, or CYP1A2 (data on file at Forma).

We report the first‐in‐human combination single‐ascending dose (SAD) and multiple‐ascending dose (MAD) study that was undertaken to characterize the safety, tolerability, pharmacokinetics (PK), and pharmacodynamics (PD) of etavopivat in healthy subjects.

## Methods

### Study Design

The study was conducted at a single site (the Medpace Clinical Pharmacology Unit [CPU], Cincinnati, Ohio). The study protocol was reviewed and approved by the local institutional review boards/independent ethics committees (Advarra, Columbia, Maryland). All study participants provided written informed consent before starting the study, and the study was conducted in accordance with the principles of the Declaration of Helsinki.

The protocol was registered at ClinicalTrials.gov (NCT03815695) and included studies designed to evaluate single and multiple doses of etavopivat in healthy subjects and patients with SCD. The studies in patients with SCD are currently ongoing and will be reported separately. Here, we report the results of single and multiple doses in healthy subjects.

### Participants

Participants were eligible for this study if they were 18 to 60 years old, with a body mass index of 18 to 33 kg/m^2^, and in general good health based on the results of medical history, a physical examination, vital signs, laboratory profile, and a 12‐lead electrocardiogram (ECG). Participants of childbearing potential could participate if they agreed to use a medically accepted contraceptive regimen during the study and for 90 days afterwards.

Key exclusion criteria were a history of any of the following: gastrointestinal surgery or resection that might alter absorption and/or excretion of orally administered drugs, or any other condition that might significantly interfere with the absorption, distribution, metabolism, or excretion of study drug; malignancy within 5 years; a history of heart problems or abnormalities; severe allergic reactions; abnormal hematologic, renal, or liver function laboratory results; alcohol abuse or dependence; or difficult venous access.

### Study Overview

**Figure 1:**
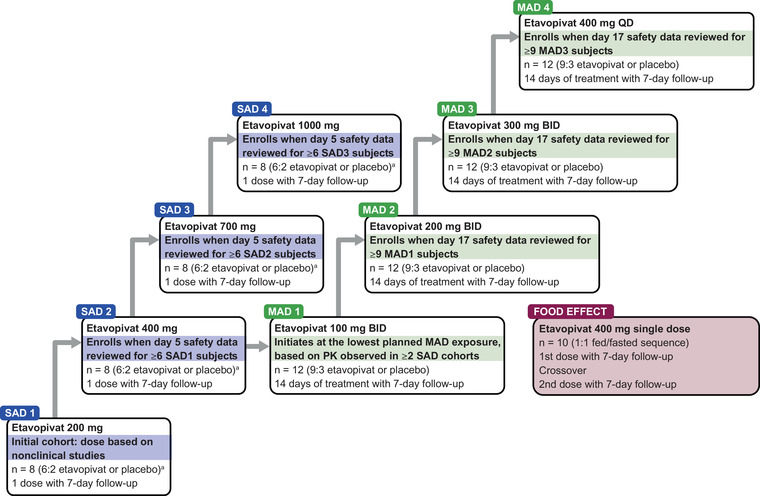
Trial design schematic. ^a^Sentinel dosing: n = 3, randomized 2:1 to etavopivat or placebo, with the remaining subjects randomized ≥48 hours later. BID, twice daily (every 12 hours); MAD, multiple‐ascending dose; PK, pharmacokinetics; QD, once daily; SAD, single‐ascending dose.

This phase 1, first‐in‐human study used a randomized (3:1), placebo‐controlled, double‐blind, SAD and MAD design, with a separate cohort to explore the effect of food on the PK profile of etavopivat. A combination approach allowing the MAD cohorts to start before all SAD cohorts finished dosing was used instead of a traditional sequential SAD/MAD evaluation (Figure 1).

#### SAD Design

The SAD study used a randomized, placebo‐controlled, double‐blind design. Four doses of etavopivat were evaluated, at escalating dose levels of 200, 400, 700, and 1000 mg. In each of the 4 dose groups, participants were randomized 3:1 to a single oral dose of either etavopivat (n = 6) or placebo (n = 2). Participants were admitted to the CPU the day before dosing. On day 1, etavopivat (or placebo) was administered in the morning, after an overnight ≥8‐hour fast, with nothing to eat for ≥4 hours after dosing. Participants stayed at the unit as inpatients for 4 days after the single‐dose etavopivat/placebo administration (days 1‐5), and returned as outpatients for an end‐of‐study visit 7 days after the study‐drug dose (on day 8).

Each cohort first enrolled a sentinel cohort (randomizing 2 participants to etavopivat and 1 participant to placebo). These participants were monitored for acute toxicity for 48 hours after dosing before the remaining 5 participants received their dose. When data were available through day 5 for ≥6 evaluable participants, subsequent cohorts could begin enrolling participants provided there were no early discontinuations related to etavopivat.

#### MAD Design

The MAD study also used a randomized, placebo‐controlled, double‐blind design that evaluated dose levels of 100, 200, or 300 mg twice daily, and 400 mg once daily. In each of these 4 dose groups, 12 participants were randomly assigned 3:1 to 14 days of etavopivat (n = 9) or placebo (n = 3). Sequential dose escalation could occur when data from ≥9 subjects within a cohort confirmed that the safety profile through day 17 was acceptable.

As with the SAD cohorts, participants were admitted to the CPU on the day before the first dose. On day 1, once‐daily etavopivat or placebo was administered in the morning after ≥1‐hour fast, with nothing to eat for ≥2 hours after dosing. For twice‐daily dosing, the afternoon dose was administered 10 to 12 hours after the morning dose, with a ≥1‐hour fast before dosing and a ≥2‐hour fast after dosing. Subjects were inpatients and received etavopivat/placebo dosing at approximately the same time each day, on days 2 to 14. Following a 3‐day washout, participants were discharged on day 17 and returned for the end‐of‐study visit 7 days after the last study‐drug dose (on day 21).

#### Food‐Effect Design

To evaluate the effect of food on etavopivat PK in healthy subjects, this study used a randomized, crossover, open‐label design. Ten participants were randomized 1:1 to receive a single dose of etavopivat 400 mg in 1 of 2 sequences (fasting/fed or fed/fasting), with a ≥8‐day washout period.

On the day before their first dose, participants were admitted to the CPU. On day 1, etavopivat was administered in the morning to participants in a fasted state (after an overnight ≥8‐hour fast) or fed state (following a high‐fat meal of 825 calories, with approximately half the calories derived from fat; specifically, 2 eggs fried in butter, 4 ounces of hash brown potatoes, 2 strips of bacon fried crisp, 2 slices of toast with butter, and 8 ounces of whole milk). Subjects were discharged on day 5 and readmitted on day 8 for the second study period when they crossed over to the opposite treatment assignment. After discharge from the second study period, subjects returned on day 16 for the end‐of‐study visit, 7 days after the last study‐drug dose.

#### Administration of Study Drug

For all participants, each study‐drug dose was administered orally (tablet formulation, 100 mg each tablet) with ≈240 mL of water.

#### Randomization and Masking

All randomization codes were generated before the start of the study. To maintain study blinding, an unblinded pharmacologist performed PK/PD analyses using dummy‐subject identifiers.

### Outcomes

The primary objectives were to assess the safety, tolerability, and PK of single and multiple doses of etavopivat in healthy adults. Secondary objectives were to evaluate the effects of etavopivat on 2,3‐DPG and ATP concentrations in RBCs and the relationship between etavopivat plasma concentration and potential effects on the QT interval in healthy subjects. The effect of food on etavopivat PK in healthy subjects was an exploratory objective.

#### Safety and Tolerability

Safety and tolerability were monitored from the time of informed consent until the end‐of‐study visit, 7 days after the last study‐drug dose. Safety was assessed by monitoring adverse events (AEs), vital signs, ECG, physical exam, and laboratory parameters. Treatment‐emergent adverse events (TEAEs) were defined as any event that was new in onset or increased in severity/frequency after the first dose of study drug until the last visit. All AEs were coded using the Medical Dictionary for Regulatory Activities version 21.0 or higher and graded according to the Common Terminology Criteria for Adverse Events version 5.0. Any clinically significant abnormalities in laboratory tests were followed until resolution or until considered stabilized by the investigator. Blood samples for the measurement of estradiol and testosterone were collected in the SAD cohorts at screening, on day –1, and 24 and 96 hours after dosing; and in the MAD cohorts at screening and on days 8, 14, and 17. Estradiol was measured by LabCorp (Burlington, North Carolina) using the Elecsys Estradiol III electrochemiluminescence immunoassay according to the manufacturer's instructions (Roche Diagnostics, Indianapolis, Indiana). The lower limit of quantitation was 25 pg/mL with a total allowable error of ≤30%. Testosterone was also measured by LabCorp using the Elecsys Testosterone II electrochemiluminescence immunoassay according to the manufacturer's instructions (Roche Diagnostics). The lower limit of quantitation was 0.120 ng/mL.

#### Cardiodynamic (Holter/Telemetry) Assessments

In addition to ECGs performed in all cohorts for safety monitoring, cardiodynamic assessments were conducted for the SAD cohorts. High‐quality ECG data were collected using continuous monitoring via telemetry or a 12‐lead Holter that was read centrally. Serial 12‐lead ECGs were extracted at prespecified time points coinciding with PK assessments to support an evaluation of the relationship between etavopivat plasma concentration and the potential effects on the QT interval.

#### Pharmacokinetics and Pharmacodynamics

For PK/PD assessments, serial blood sampling to determine the plasma concentrations of etavopivat was performed before dosing, on day 1 (all cohorts) and day 14 (MAD cohorts), for up to 72 hours after the last dose, and at the end‐of‐study visit on day 8 (SAD cohorts) or day 21 (MAD cohorts). Urine samples were collected for the SAD cohorts only, before dosing and up to 96 hours after the last dose.

Etavopivat plasma concentrations were analyzed using a validated liquid chromatography–tandem mass spectrometry method (Charles River Laboratories, Shrewsbury, Massachusetts). Stable isotope‐labeled analog ^13^C6‐etavopivat was used as an internal standard. Using plasma samples in 25 μL of dipotassium ethylenediaminetetraacetic acid (K_2_‐EDTA), acetonitrile was used for the extraction of etavopivat and the internal standard. Extracted samples were analyzed using a liquid chromatography system coupled with an API 5500 mass spectrometer (AB Sciex, Redwood City, California). Chromatographic separation was performed on a 2.1 × 50 mm, 3‐μm ACE 3 C18 column kept at 40^o^C. Mobile phase A was water : acetonitrile : formic acid (95:5:0.1, v:v:v) and mobile phase B was acetonitrile:methanol:formic acid (50:50:0.1, v:v:v). The mass transitions monitored for etavopivat and ^13^C6‐etavopivat were m/z 458.2>310.1 and 464.2>310.1 and were detected using a multiple reaction monitoring scan mode with positive‐ion electrospray ionization. The analytical range was 1.0 to 1000 ng/mL, and the percent coefficient of variation (%CV) for all intra‐ and interbatch analyses was <15%. The lower limit of quantitation for etavopivat was 1.00 ng/mL.

The concentrations of ATP and 2,3‐DPG were measured simultaneously using a liquid chromatography–tandem mass spectrometry method (Charles River Laboratories) in K_2_­EDTA–anticoagulated whole blood samples. Analysis was performed using an API 5500 triple‐quadrupole mass spectrometer (AB Sciex) coupled with an Agilent 1200 series high‐performance liquid chromatography system (Agilent 1260 Infinity Bin Pump and G1379B Degasser; Agilent, Santa Clara, California) and a CTC‐PAL autosampler (Thermo Fisher Scientific, Waltham, Massachusetts). Analyst 1.6.2 (AB Sciex) and Aria MX software (Thermo Fisher Scientific) were used for instrument control, data acquisition, and data analysis. Calibration standards (25.0–1500 μg/mL), quality controls, and study samples were thawed on wet ice and vortex‐mixed for ≈2 minutes before being pipetted. Because ATP and 2,3‐DPG are endogenous molecules present in whole blood at high levels, the low, middle, and high quality‐control calibration standards were prepared in deionized water (surrogate matrix). Whole blood samples (15 μL) were spiked with stable isotope–labeled internal standard (^13^C_10_, ^15^N_5_‐ATP, and D_3_‐2,3‐DPG), processed by protein precipitation extraction, and analyzed using ZIC‐pHILIC separation with Turbo Ion Spray tandem mass spectrometry detection. The high‐performance liquid chromatography column (2.1 × 50 mm SeQuant ZIC‐pHILIC) was kept at 40^o^C. Negative (M‐H)^−^ ions for ATP and 2,3‐DPG and their respective internal standards, ^13^C_10_, ^15^N_5_‐ATP, and D_3_‐2,3‐DPG, were monitored in multiple reaction monitoring mode (mass transitions monitored were m/z 506.0>159.0 and 521.0>159.0 for ATP and ^13^C_10_, ^15^N_5_‐ATP, and 265.0>166.8 and 268.0>169.8 for 2,3‐DPG and D_3_‐2,3‐DPG). Mobile phase A was 10 mM ammonium acetate in water, pH 8.5; and mobile phase B was acetonitrile : mobile phase A solution (5:95, v:v). Analyte to internal standard peak area ratios for the standards were used to create a quadratic calibration curve using 1/x^2^ weighted least‐squares regression analysis. For both analytes, the overall accuracy of the method was within ±10.5% (% relative error), and the intra‐ and interassay precision %CV was <7%.

The effect of a 2,3‐DPG reduction in RBCs on Hb‐oxygen affinity was compared for the SAD and MAD cohorts using P_50_. Hb‐oxygen affinity was measured before dosing and 24 hours after the last dose (ie, at 24 hours for the SAD cohorts and at day 15 for the MAD cohorts) by oxyhemoglobin dissociation curves collected using a TCS HEMOX Analyzer^17^ (TCS Scientific Corp., New Hope, Pennsylvania). Whole blood samples were collected in K_2_­EDTA tubes, diluted 50‐fold into prewarmed (37^o^C) TES sodium salt buffer (Sigma Aldrich, St. Louis, Missouri) containing 20 μL of bovine serum albumin‐20 additive A and 10 μL antifoaming agent‐25 per 5 mL of buffer, and oxygenated using compressed air. Measurement started at an oxygen pressure of 145 mm Hg following replacement of air with nitrogen, and was stopped automatically at an oxygen pressure of 1.9 mm Hg.

### Statistical Analyses

The sample size for this phase 1 study was considered adequate to evaluate the safety, tolerability, and PK/PD of etavopivat; no formal power calculations were performed. The safety analysis set included all participants who received ≥1 dose of any study treatment (etavopivat or placebo). The PK analysis set included all participants who received etavopivat and had sufficient concentration data to enable the calculation of ≥1 PK parameter without protocol violations that had the potential to affect the PK concentrations. The PD analysis set included all safety analysis set participants who had baseline and ≥1 postdose PD assessment. The corrected QT interval (QTc) analysis set included all safety analysis set participants with telemetry ECG measurements of QT corrected by Fridericia's formula (QTcF) at baseline and on treatment with ≥1 postdose time point with a valid value for ΔQTcF (change from baseline in QTcF). The food‐effect analysis set included participants in the food‐effect cohort who received both planned study‐drug doses (with/without food).

Demographics, baseline characteristics, safety, and PK/PD data were summarized separately for each cohort using descriptive statistics. Participants who received the placebo were pooled together for analyses.

The relationship between PK concentration and time‐matched change from baseline QTcF adjusted for placebo (ΔΔQTcF) was evaluated using a linear mixed‐effects model.^18^ ΔΔQTcF was calculated as ΔΔQTcF (*i,j*) = ΔQTcFf (*i,j*) – mean ΔQTcFp (*k*), where ΔQTcFf (*i,j*) was the change from baseline in QTcF for subject *i* at time *j* for subjects treated with etavopivat, and mean ΔQTcFp (*k*) is the average change from baseline in QTcF at time *j* for the pooled placebo group. The model‐predicted ΔΔQTcF, with a 2‐sided 90%CI, was derived corresponding to the geometric mean of the maximum observed plasma concentration (C_max_) from each SAD dose cohort.

Standard noncompartmental methods were used to calculate PK parameters, using actual sampling times, with Phoenix WinNonlin 8.0 (Certara, Princeton, New Jersey). The time to maximum observed plasma concentration (t_max_) and C_max_ were taken directly from individual participants’ data. The area under the plasma concentration–time curve (AUC) was calculated using the linear‐log trapezoidal method. Fed/fasted PK results were compared using an analysis of variance model for the natural log‐transformed C_max_ and AUC.

Clinical PK and PD data were modeled using a simultaneous population PK/PD approach utilizing Phoenix NLME software (Certara). The PK data were fit using a 2‐compartment model with first‐order absorption. The PD data were fit using a basic indirect‐response model with a simulation of loss or production function for 2,3‐DPG and ATP, respectively. Population simulations using the final model were performed to predict the steady‐state exposure–response relationship at different doses.

Relevant PD correlations were evaluated using the coefficient of determination from the general linear regression. Pre‐ versus postdose changes in Hb‐oxygen affinity were compared in participants who received etavopivat using the Wilcoxon signed‐rank test for nonparametric paired data, using HEMOX Analyzer Software (TCS Scientific Corp.). Statistical analyses were performed at a 2‐sided significance level of 0.05. Analyses were considered exploratory and not adjusted for multiplicity.

Statistical analyses were performed using SAS version 9.3 or higher (SAS Institute, Cary, North Carolina). Prism version 9.0 (GraphPad Software, San Diego, California) was used for linear regression.

## Results

### Participants

The study was completed between December 2018 and May 2019. A total of 90 healthy subjects were included: 32 in the SAD cohorts (etavopivat, n = 24; placebo, n = 8), 48 in the MAD cohorts (etavopivat, n = 36; placebo, n = 12), and 10 in the food‐effect cohort. All subjects received study treatment as per the randomization sequence, completed the study, and were included in analyses (Figure S2). Demographics and participant characteristics were balanced across the cohorts (Table S1).

### Safety and Tolerability

Across all study cohorts, there were no serious AEs or TEAEs that led to the discontinuation of etavopivat. Dose‐limiting toxicity was not identified at any of the doses in the study. In the healthy‐subject SAD cohorts, 6 of 32 subjects (19%) had ≥1 TEAE (Table S2). In the placebo arm, 1 of 8 subjects (13%) had 1 TEAE (gastroenteritis). In the etavopivat group, 5 of 24 subjects (21%) reported a total of 6 TEAEs (abdominal pain, increased amylase, headache, increased lipase, urethral discharge, and ventricular arrhythmia). Of these TEAEs, the single events of headache and ventricular arrhythmia were considered to be possibly related to study drug. The episode of asymptomatic ventricular arrhythmia was an incidental finding of 4 beats noted on telemetry and was considered not clinically significant after a comprehensive blinded cardiac evaluation.

Across the MAD cohorts, 18 of 48 subjects (38%) had ≥1 TEAE, including 15 of 36 (42%) who received etavopivat and 3 of 12 (25%) who received placebo (Table S3). In these cohorts, the most common TEAE was headache, reported in 10 of 36 (28%) etavopivat‐treated subjects and 2 of 12 (17%) placebo‐group subjects. All other TEAEs in the MAD cohorts were reported in 1 subject each (Table S3). Treatment‐related TEAEs for the MAD cohorts included headache in 4 of 36 subjects (11%), and palpitations and somnolence in 1 subject each (3%).

In the food‐effect cohort, 2 of 10 subjects (20%) reported TEAEs, which were mild and considered not related to study drug. In the fasted/fed sequence, 1 subject had an event of skin abrasion (fed period) and in the fed/fasted sequence, 1 subject had an event of rhinitis (fasted period).

Across all study cohorts, most TEAEs were mild (grade 1) or moderate (grade 2) in severity, and all events resolved without sequelae. One participant who received a single dose of etavopivat 1000 mg was reported to have a transient, asymptomatic, grade 3 event of increased lipase. When the subject's back‐up laboratory sample was reassessed independently, no lipase increase was observed.

Vital signs and laboratory tests did not reveal any notable pattern of change following study‐drug administration. There were no observed effects on testosterone or estradiol levels, with changes in males in the MAD cohorts showing no meaningful differences from baseline (Figure S3). No abnormal, clinically significant 12‐lead ECG findings were reported.

#### Cardiodynamic (Holter/Telemetry) Assessments

For the SAD cohorts with high‐quality ECG data, no QT‐prolongation effects of etavopivat were observed. There were no heart rate effects nor evidence of a nonlinear relationship between etavopivat concentration and change in QTcF interval. Evaluation of geometric mean plasma C_max_ values across dose levels confirmed that an exposure margin of >2‐fold was achieved between the predicted highest clinical exposure and the highest dose tested (1000 mg). Mean (90%CI) for the model‐predicted placebo‐adjusted change from baseline in QTcF interval at the 1000‐mg dose level was –1.06 (–5.91, 3.79) confirming that the upper bound of the 2‐sided 90%CI for the QTc effect of etavopivat treatment, as estimated by the exposure–response analysis, was <10 milliseconds at the highest exposure studied (Figure S4 and Table S4).

### Pharmacokinetics

**Figure 2:**
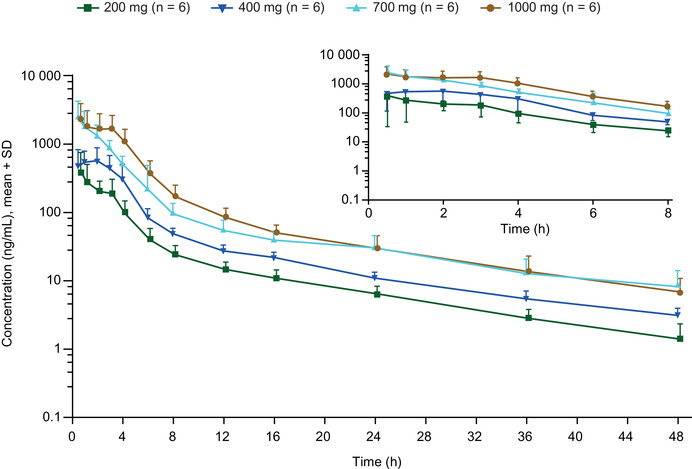
Etavopivat plasma concentrations following single doses (200–1000 mg) in healthy subjects. Data points are offset for clarity. Inset shows the interval from 0 to 8 hours on an extended time scale. Concentrations below the LLOQ were set to zero. LLOQ, lower limit of quantitation; SD, standard deviation.

Plasma etavopivat concentrations following oral administration of a single dose under fasted conditions are shown in Figure 2. Etavopivat was rapidly absorbed, with a median t_max_ range of 0.5 to 1.5 hours and a biphasic elimination profile with the mean estimated terminal elimination t_1/2_ ranging from 10.6 to 13.8 hours. Apparent clearance was consistent across dose levels (Table 1; Table S5). Dose‐normalized C_max_ and AUC increased with increasing doses ≥700‐mg, indicating greater‐than‐dose‐proportional increases in exposure at the highest doses tested. Intersubject variability in etavopivat plasma concentrations was moderate (AUC from time 0 to the last value above the limit of quantification %CV for geometric mean was 40.6, 23.6, 30.5, and 45.8 for the 4 dose groups). The fraction of unchanged etavopivat excreted in urine was ≈1% (for the 4 dose groups, mean fraction excreted unchanged in urine values ranged from 0.8% to 1.7%). Renal clearance was consistent across the dose range (mean renal clearance values ranged from 1.0 to 1.5 L/h).

**Table 1:** Single‐Dose Etavopivat PK Values in Healthy Subjects

	Etavopivat Dose			
	200 mg	400 mg	700 mg	1000 mg
Parameter^a^	(n = 6)	(n = 6)	(n = 6)	(n = 6)
**Plasma**				
AUC_0–24h_, ng·h/mL	1214 (573)	2635 (635)	6707 (1945)	9137 (4442)
AUC_0–last_, ng·h/mL	1297 (582)	2818 (676)	7217 (2134)	9586 (4395)
AUC_0–inf_, ng·h/mL	1320 (581)	2847 (672)	7254 (2134)	9618 (4402)
C_max_, ng/mL	447 (312)	786 (166)	2652 (1484)	2663 (1118)
t_max_, h^b^	0.50 (0.5, 3.0)	1.50 (0.5, 4.0)	0.53 (0.5, 6.0)	0.51 (0.5, 3.0)
t_1/2_, h	11.4 (3.5)	12.9 (2.6)	13.8 (3.9)	10.6 (2.4)
CL/F, L/h	171.1 (56.8)	146.7 (31.9)	103.8 (30.3)	121.4 (47.0)
**Urine**				
CLr, L/h	1.5 (0.5)	1.0 (0.5)	1.4 (0.2)	1.1 (0.3)
f_e_, %	1.7 (0.4)	0.8 (0.2)	1.3 (0.3)	0.9 (0.3)

AUC, area under the concentration–time curve; AUC_0–24/last_, AUC from time 0 until the 24 h/last time point; AUC_0–inf_, AUC from time 0 extrapolated to infinity; CL/F, apparent clearance; CLr, renal clearance; C_max_, maximum plasma concentration; f_e_, fraction excreted unchanged in urine; PK, pharmacokinetic; t_1/2_, terminal elimination half‐life; t_max_, time to maximum concentration.

^a^ Values are arithmetic mean (SD) except where indicated.

^b^ Median (minimum, maximum).

**Figure 3:**
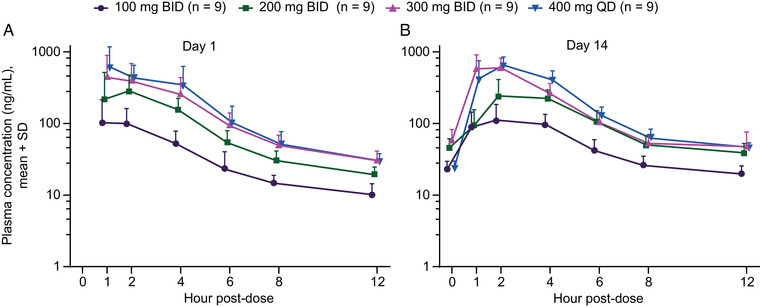
Etavopivat plasma concentrations on (A) day 1 and (B) day 14 following multiple doses in healthy subjects. Data points are offset for clarity. Concentrations below the LLOQ were set to zero. BID, twice daily; LLOQ, lower limit of quantitation; QD, once daily; SD, standard deviation.

Following multiple dosing under modified fasted conditions, etavopivat PK displayed moderate intersubject variability and exposure increased in a dose‐proportional manner (Figure 3). Exposure on day 1 was similar to that on day 14. The C_max_ ratio ranged from 0.94 to 1.54, and AUC ratio ranged from 1.24 to 1.46 across dose levels (Table 2; Table S6).

**Table 2:** Multiple‐Dose Etavopivat Plasma PK in Healthy Subjects

		Etavopivat Dose			
		100 mg BID	200 mg BID	300 mg BID	400 mg QD
Parameter^a^	Day	(n = 9)^b^	(n = 9)^c^	(n = 9)^b^	(n = 9)^d^
AUC_0–tau_, ng·h/mL	1	494 (162)	1088 (403)	14709 (569)	2566 (776)
AUC_0–tau_, ng·h/mL	14	588 (221)	1138 (506)	2410 (167)	3018 (626)
Ratio AUC_0–tau_		1.46 (0.08)	1.36 (0.58)	1.27 (0.12)	1.24 (0.33)
AUC_0–last_, ng·h/mL	1	451 (147)	1131 (400)	1841 (911)	2485 (765)
AUC_0–inf_, ng·h/mL	1	555 (180)	1203 (438)	1601 (618)	2769 (786)
C_max_, ng/mL	1	148 (93)	408 (229)	594 (371)	837 (395)
C_max_, ng/mL	14	148 (55)	313 (97)	733 (216)	694 (185)
Ratio C_max_		1.22 (0.51)	0.94 (0.48)	1.54 (0.73)	1.04 (0.61)
t_max_, h^e^	1	1.00 (1.0, 4.0)	2.00 (1.0, 4.0)	2.00 (1.0, 4.0)	1.00 (1.0, 4.0)
t_1/2_, h	1	5.1 (0.7)	4.4 (1.8)	3.8 (1.3)	10.8 (2.4)
CL/F, L/h	1	198.4 (70.1)	191.4 (87.7)	225.3 (121.9)	155.4 (45.5)
CL/F_SS_, L/h	14	184.3 (57.2)	196.6 (70.9)	124.9 (8.4)	137.6 (28.0)

AUC, area under the concentration–time curve; AUC_last_, AUC from time 0 until the last time point; AUC_0–inf_, AUC from time 0 extrapolated to infinity; AUC_0–tau_, AUC from time 0 to the end of the dosing period (that is, 0‐12 for twice daily and 0‐24 for once daily); BID, twice daily; CL/F, apparent clearance; CL/F_SS_, apparent clearance at steady state; C_max_, maximum plasma concentration; PK, pharmacokinetic; QD, once daily; SD, standard deviation; t_1/2_, terminal elimination half‐life; t_max_, time to maximum concentration.

^a^ Values are arithmetic mean (SD) except where indicated.

^b^ n = 6 for day 1 AUC_0–tau_, day 1 AUC_0–inf_, t_1/2_, and CL/F; n = 3 for day 14 AUC_0–tau_, CL/F_SS_, and ratio AUC_0–tau_.

^c^ n = 8 for day 1 AUC_0–tau_, day 1 AUC_0–inf_, t_1/2_, and CL/F; n = 3 for day 14 AUC_0–tau_, CL/F_SS_, and ratio AUC_0–tau_.

^d^ n = 8 for day 1 AUC_0–tau_, day 1 AUC_0–inf_, t_1/2_, CL/F, and day 14 ratio AUC_0–tau_.

^e^ Median (minimum, maximum).

In the food‐effect cohort, there was a slight delay in absorption following a 400‐mg single dose of etavopivat under fed vs fasted conditions, with median (range) t_max_ of 3.0 (0.8‐6.0) hours versus 1.0 (0.5‐6.0) hours under fasted conditions (Figure S5; Table S7). Overall, statistical analysis of plasma PK parameters indicated that the effects of food on etavopivat exposure were minimal and not clinically meaningful. More specifically, 90%CI for C_max_ geometric mean ratio ranged from 60.3% to 111.4% and AUC_inf_ geometric mean ratio ranged from 110.0% to 128.1% (Table S8).

### Pharmacodynamics

**Figure 4:**
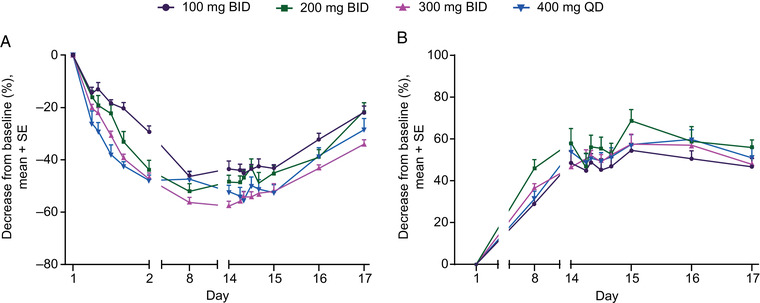
Change from baseline in (A) 2,3‐DPG and (B) ATP in healthy subjects receiving multiple doses of etavopivat for 14 days. 2,3‐DPG, 2,3‐diphosphoglycerate; ATP, adenosine triphosphate; BID, twice daily; QD, once daily; SE, standard error.

Blood 2,3‐DPG levels decreased after single and multiple doses of etavopivat. Maximal decrease was observed ≈24 hours post‐dose and was sustained for >1 day following the last dose (Figure 4A). Blood ATP concentrations increased after repeat dosing with etavopivat. Maximal increase was observed between days 8 and 14 of repeated dosing and this increase was sustained for >3 days following the last dose (Figure 4B). Modeling of the predicted PD response in the RBCs of healthy subjects was used to define an exposure–response relationship (Figure S6). These analyses indicated that a daily etavopivat dose of ≥150 mg achieved the maximal response for ATP and a daily dose of ≥400 mg achieved the maximal response for 2,3‐DPG.

**Figure 5:**
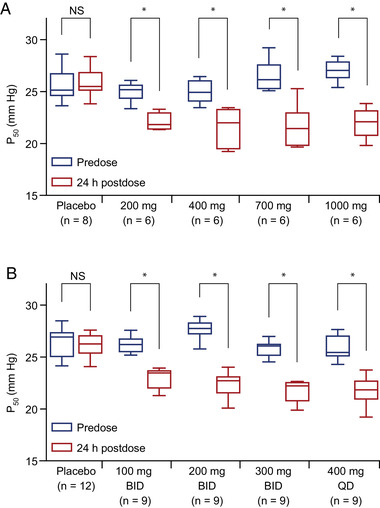
Changes in hemoglobin‐oxygen affinity in RBCs of healthy subjects with etavopivat (A) single dose and (B) multiple doses. *All *P* < .05. *P* values based on Wilcoxon matched‐pairs signed‐rank test. Horizontal lines show the median values, the boxes the 25th to 75th percentiles, and the whiskers show the minimum to maximum values. BID, twice daily; NS, not significant; P_50_, partial pressure of oxygen at which hemoglobin‐oxygen saturation of 50% is achieved; QD, once daily; RBC, red blood cell.

**Figure 6:**
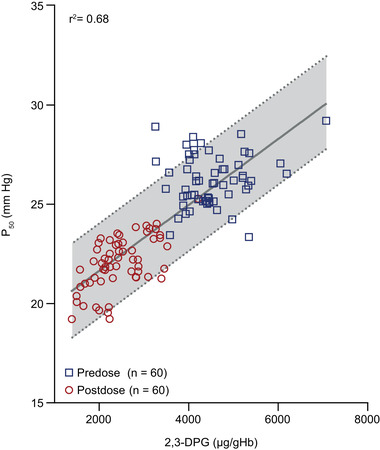
Correlation between 2,3‐DPG concentrations and hemoglobin‐oxygen affinity. Linear regression: r^2^ is the coefficient of determination and the shaded regions show the 90% prediction band. 2,3‐DPG, 2,3‐diphosphoglycerate; P_50_, partial pressure of oxygen at which hemoglobin‐oxygen saturation of 50% is achieved.

Increased Hb‐oxygen affinity (decreased P_50_) was observed at all etavopivat doses tested (Figure 5). Change in Hb‐oxygen affinity correlated with a reduction in 2,3‐DPG, with improved Hb‐oxygen affinity observed at lower levels of 2,3‐DPG (Figure 6).

## Discussion

This first‐in‐human combined SAD and MAD study evaluated the safety, tolerability, and PK/PD of etavopivat in healthy subjects. Following administration of single and multiple doses, no dose‐limiting toxicity was observed with any of the doses tested, and etavopivat was well tolerated, with no AEs leading to discontinuation of the study drug. Most AEs were mild (grade 1) in severity and most were considered unrelated to the study drug. For the multiple‐dose cohorts, headache was the most common AE, although there was no apparent relationship between etavopivat dose and the incidence of headache. Notably, headache has also been reported as the most common AE for another PKR activator, mitapivat, in healthy subjects and in patients with pyruvate kinase deficiency.^19^, ^20^ No abnormal laboratory values were reported, and there was no apparent aromatase inhibition, with no observed effects on the production or metabolism of estradiol or testosterone.

The PK profile of etavopivat following single‐ascending oral doses confirmed that etavopivat was rapidly absorbed, with a median t_max_ range of 0.5 to 1.5 hours. A multiexponential decline in plasma etavopivat concentrations was observed following C_max_, with concentrations declining rapidly up to ≈8 hours after dosing followed by a much slower decline between 12 and 36 hours after dosing. Mean elimination t_1/2_ ranged from 10.6 to 13.8 hours in the single‐dose cohorts and from 3.8 to 10.8 hours in the multiple‐dose cohorts. This difference in t_1/2_ estimates was attributed to the fact that the terminal elimination phase was not fully measurable due to the shorter dosing interval in the twice‐daily cohorts.

Etavopivat exposure increased in a greater‐than‐dose‐proportional manner at doses ≥700 mg. Following daily dosing of etavopivat for 14 days, there was no significant drug accumulation between day 1 and steady state. Although mean C_max_ and AUC ratios were similar across dose levels, there appeared to be a large difference in mean AUC over the dosing interval (AUC_0‐tau_) on day 1 compared with day 14 for the 300 mg twice‐daily dose group. This inconsistency is likely due to limited estimates for AUC_0‐tau_ and moderate PK variability. Given the limitations described above in characterizing the terminal elimination phase, AUC_0‐tau_ could not be calculated for all subjects and mean AUC_0‐tau_ was based on only 3 participants (of 9 in total). Renal clearance was consistent across the dose range, confirming that renal excretion plays a minor role in the systemic elimination of etavopivat.

Analyses of PK/PD data were performed to assess the PK of etavopivat and the PK/PD relationship of etavopivat to 2,3‐DPG and ATP in healthy subjects and confirmed time delays between peak drug levels and peak PD effects. Based on the single‐dose plasma PK profile, fractionated dosing, twice daily (every 12 hours), was selected for the 14‐day MAD evaluations to remain within the established linear exposure range and potentially maximize target coverage and the resulting PD response. Etavopivat exhibited linear and time‐independent PK, and evaluation of the PD profiles showed decreased 2,3‐DPG and increased ATP occurring after C_max_. The PD effects were durable, continuing for ≈2 days after the last dose, and as such, potentially support a once‐daily schedule. This was confirmed in the fourth MAD cohort, which evaluated etavopivat 400 mg once daily for 14 days. Based on PK/PD modeling, it was predicted that maximal effects for 2,3‐DPG and ATP could be achieved at doses ≥400 mg and ≥150 mg once daily, respectively, and on this basis, a once‐daily schedule was recommended for evaluation in patients with SCD.

Administration of etavopivat also resulted in increased Hb‐oxygen affinity (as measured by a reduction in P_50_, the partial pressure of oxygen at which 50% of Hb is saturated with oxygen). Although these results in healthy volunteers cannot be directly correlated with potential clinical benefits in patients with SCD, it was notable that significant improvements were observed at all dose levels of etavopivat. In addition, changes in Hb‐oxygen affinity correlated with a reduction in 2,3‐DPG levels, suggesting that the action of etavopivat will alter biological activity.

The study also included a robust QT‐prolongation risk assessment. As outlined in regulatory guidelines, concentration–QT modeling is a reasonable substitute for a dedicated thorough‐QT trial to classify the risk of QTc prolongation and to determine if intensive ECG safety monitoring should be included in a pivotal trial.^21^ Therefore, high‐quality ECG data were collected for all SAD cohorts to facilitate an evaluation of the relationship between etavopivat plasma concentration and potential effects on the QT interval. No QTc prolongation effects were observed for etavopivat and the upper bound of the 90%CI of the mean predicted ΔQTcF remained below 10 milliseconds at all doses of etavopivat, despite exposure at the 1000‐mg dose being >2‐fold above the predicted therapeutic exposure. These results indicate that routine ECG monitoring is suitable for future trials of etavopivat.

The exploratory evaluation of the effects of food was limited to some extent by the small sample size; however, there appeared to be no clinically meaningful effects on the PK of etavopivat following a high‐fat, high‐calorie meal. Based on these results, this tablet formulation of etavopivat can be administered irrespective of food in future trials.

## Conclusions

This phase 1 study of etavopivat in healthy subjects established that multiple doses of etavopivat are well tolerated at dose levels demonstrated to have PD activity, as well as confirming that etavopivat can be taken as a once‐daily regimen with or without food. These results provide the foundation for further investigation of etavopivat in patients with SCD, for whom it may be anticipated that a sustained reduction in 2,3‐DPG combined with an increase in ATP production may reduce hemolysis and ameliorate anemia. Studies are ongoing to evaluate the effect of etavopivat on RBC metabolism, inflammation, and coagulation, as well as further evaluate the safety, PK, PD, and clinical activity of once‐daily etavopivat in patients with SCD (The Hibiscus Study; NCT04624659).

## Conflicts of Interest

This study was funded by Forma Therapeutics, Inc., Watertown, Massachusetts, the manufacturer of etavopivat. S.F., P.S., J.G., M.D.R., E.W., and P.K. are employees of Forma Therapeutics. L.V. and K.L. are employees of Medpace Clinical Pharmacology, which was contracted by Forma Therapeutics for the clinical studies reported here. D.G.K. reports no conflicts of interest. T.A.K. reports consultancy for Agios Pharmaceuticals, and research funding from Agios Pharmaceuticals, Forma Therapeutics, and National Institutes of Health National Heart, Lung, and Blood Institute. All authors had full access to the study data and were involved in the analysis, interpretation, and preparation of this report. All authors were responsible for the decision to submit the manuscript for publication.

## Supporting information

Supporting information

## Data Availability

Individual participant data (including data dictionaries) will not be available.
