# Supplementary material for: Safety, Pharmacokinetics, and Pharmacodynamics of Etavopivat (FT‐4202), an Allosteric Activator of Pyruvate Kinase‐R, in Healthy Adults: A Randomized, Placebo‐Controlled, Double‐Blind, First‐in‐Human Phase 1 Trial
Source: Clin Pharmacol Drug Dev. 2022 Jan 12;11(5):654–65. doi: 10.1002/cpdd.1058 (PMC9306898; doi:10.1002/cpdd.1058)
Supplement: Supplementary file 1 — Supporting information [file CPDD-11-654-s001.docx]

# Supplemental Information

#### **Supplementary Table S1.** Participant Demographics and Baseline Characteristics

|  | **Single-Dose Cohorts** | | | **Multiple-Dose Cohorts** | | | **Food-Effect Cohort** | |
| --- | --- | --- | --- | --- | --- | --- | --- | --- |
|  | Placebo | Etavopivat | Placebo | | Etavopivat | Fasted/Fed | | Fed/Fasted |
|  | (n = 8) | (n = 24) | (n = 12) | | (n = 36) | (n = 5) | | (n = 5) |
| Age, y, mean (SD) | 40.8 (6.1) | 44.5 (11.3) | 44.8 (12.1) | | 44.8 (11.1) | 48.8 (3.9) | | 37.8 (8.6) |
| Male sex, n (%) | 6 (75) | 14 (58) | 6 (50) | | 22 (61) | 3 (60) | | 4 (80) |
| Race, n (%) |  |  |  | |  |  | |  |
| White | 6 (75) | 10 (42) | 5 (42) | | 20 (56) | 3 (60) | | 0 |
| Black | 2 (25) | 14 (58) | 4 (33) | | 13 (36) | 2 (40) | | 3 (60) |
| Other/ Multiple^a^ | 0 | 0 | 3 (25) | | 3 (8) | 0 | | 2 (40) |
| Weight, kg, mean (SD) | 79 (15) | 81 (14) | 73 (13) | | 80 (9) | 68 (13) | | 92 (12) |
| BMI, kg/m^2^, mean (SD) | 26.9 (3.2) | 27.1 (4.1) | 25.1 (3.6) | | 26.7 (2.7) | 26.8 (2.4) | | 30.0 (2.8) |

BMI, body mass index; SD, standard deviation.

^a^ Other/Multiple includes Asian, American Indian or Alaska Native, Native Hawaiian or Other Pacific Islander.

#### **Supplementary Table S2.** Treatment-Emergent Adverse Events in Healthy Subjects Receiving a Single Dose of Etavopivat or Placebo

|  | **Placebo** | **Etavopivat** | | | | |
| --- | --- | --- | --- | --- | --- | --- |
|  | Pooled | 200 mg | 400 mg | 700 mg | 1000 mg | Total |
|  | (n = 8) | (n = 6) | (n = 6) | (n = 6) | (n = 6) | (n = 24) |
| Any TEAE, n (%) | 1 (13) | 2 (33) | 1 (17) | 0 | 2 (33) | 5 (21) |
| Abdominal pain | 0 | 1 (17) | 0 | 0 | 0 | 1 (4) |
| Amylase increased | 0 | 0 | 0 | 0 | 1 (17) | 1 (4) |
| Gastroenteritis | 1 (13) | 0 | 0 | 0 | 0 | 0 |
| Headache | 0 | 1 (17) | 0 | 0 | 0 | 1 (4) |
| Lipase increased | 0 | 0 | 0 | 0 | 1 (17) | 1 (4) |
| Urethral discharge | 0 | 0 | 0 | 0 | 1 (17) | 1 (4) |
| Ventricular arrhythmia | 0 | 0 | 1 (17) | 0 | 0 | 1 (4) |

TEAE, treatment-emergent adverse event.

All TEAEs were grade 1 except for grade 2 gastroenteritis, grade 2 amylase increased, and grade 3 lipase increased. When the subject’s back-up laboratory result for lipase increased sample was re-assessed independently, no lipase increase was observed.

#### **Supplementary Table S3.** Treatment-Emergent Adverse Events in Healthy Subjects Receiving Etavopivat or Placebo for 14 Days

|  | **Placebo** | **Etavopivat** | | | | |
| --- | --- | --- | --- | --- | --- | --- |
|  | Pooled | 100 mg BID | 200 mg BID | 300 mg BID | 400 mg QD | Total |
|  | (n = 12) | (n = 9) | (n = 9) | (n = 9) | (n = 9) | (n = 36) |
| Any TEAE, n (%) | 3 (25) | 5 (56) | 4 (44) | 3 (33) | 3 (33) | 15 (42) |
| Conjunctivitis | 0 | 0 | 0 | 0 | 1 (11) | 1 (3) |
| Diarrhea | 1 (8) | 0 | 1 (11) | 0 | 0 | 1 (3) |
| Dyspnea | 0 | 1 (11) | 0 | 0 | 0 | 1 (3) |
| Eye irritation | 0 | 0 | 0 | 1 (11) | 0 | 1 (3) |
| Folliculitis | 0 | 0 | 0 | 1 (11) | 0 | 1 (3) |
| Headache | 2 (17) | 4 (44) | 3 (33) | 2 (22) | 1 (11) | 10 (28) |
| Influenza-like illness | 0 | 0 | 1 (11) | 0 | 0 | 1 (3) |
| Muscle spasms | 1 (8) | 0 | 0 | 0 | 0 | 0 |
| Musculoskeletal pain | 0 | 0 | 0 | 1 (11) | 0 | 1 (3) |
| Nasal congestion | 0 | 0 | 0 | 0 | 1 (11) | 1 (3) |
| Neck pain | 0 | 0 | 0 | 0 | 1 (11) | 1 (3) |
| Oropharyngeal pain | 0 | 1 (11) | 0 | 0 | 0 | 1 (3) |
| Palpitations | 0 | 1 (11) | 0 | 0 | 0 | 1 (3) |
| Rash | 0 | 0 | 1 (11) | 0 | 0 | 1 (3) |
| Rhinitis | 0 | 0 | 1 (11) | 0 | 0 | 1 (3) |
| Somnolence | 0 | 1 (11) | 0 | 0 | 0 | 1 (3) |
| Upper RTI | 1 (8) | 0 | 0 | 0 | 0 | 0 |

BID, twice daily; QD, once daily; RTI, respiratory tract infection; TEAE, treatment-emergent adverse event.

All TEAEs were grade 1.

#### **Supplementary Table S4.** Placebo-Adjusted Change from Baseline in QTcF Interval (ΔΔQTcF) in Healthy Subjects Receiving a Single Dose of Etavopivat: Linear Mixed-Effect Model

|  | **Geometric Mean Etavopivat C_max_ (ng/mL)** | **ΔΔQTcF (ms)** | **90% CI (ms)** |
| --- | --- | --- | --- |
| 200 mg | 379 | –2.35 | (–3.34, –1.36) |
| 400 mg | 769 | –2.11 | (–3.52, –0.70) |
| 700 mg | 2204 | –1.22 | (–5.55, 3.11) |
| 1000 mg | 2452 | –1.06 | (–5.91, 3.79) |

CI, confidence interval; ms, milliseconds; PK, pharmacokinetic; QTcF, QT corrected by Fridericia’s formula.

A linear mixed-effect model was used with the individual placebo-adjusted QTcF change from baseline (ΔΔQTcF) as the response variable, time-matched etavopivat plasma concentration as a covariate, and a random slope and intercept per subject. A compound symmetry covariance structure matrix were used to estimate the intercept-slope covariance. Placebo PK concentrations were set to zero. For etavopivat, post-dose PK concentrations below the lower limit of quantification were imputed to zero.

#### **Supplementary Table S5.** Single-Dose Etavopivat PK Values in Healthy Subjects [Geometric Mean (Geometric %CV) Except Where Indicated]

|  | **Etavopivat Dose** | | | |
| --- | --- | --- | --- | --- |
|  | 200 mg | 400 mg | 700 mg | 1000 mg |
| **Parameter^a^** | (n = 6) | (n = 6) | (n = 6) | (n = 6) |
| **Plasma** |  |  |  |  |
| AUC_0–24h_, ng∙h/mL | 1127 (42) | 2574 (24) | 6468 (31) | 8331 (49) |
| AUC_0–last_, ng∙h/mL | 1210 (41) | 2754 (24) | 6956 (31) | 8827 (46) |
| AUC_0–inf_, ng∙h/mL | 1235 (40) | 2785 (23) | 6995 (30) | 8860 (46) |
| C_max_, ng/mL | 379 (65) | 769 (23) | 2204 (84) | 2452 (48) |
| T_max_, h^b^ | 0.50 (0.5, 3.0) | 1.50 (0.5, 4.0) | 0.53 (0.5, 6.0) | 0.51 (0.5, 3.0) |
| t_½_, h | 11.0 (27.2) | 12.7 (20.8) | 13.3 (34.3) | 10.4 (23.0) |
| CL/F, L/h | 161.9 (39.7) | 143.6 (23.2) | 100.1 (30.3) | 112.9 (45.7) |
| **Urine** |  |  |  |  |
| CLr, L | 1.4 (31.5) | 0.9 (53.8) | 1.3 (13.7) | 1.0 (38.0) |
| f_e_, % | 0.8 (26.2) | 0.6 (44.9) | 1.2 (23.1) | 0.8 (29.2) |

AUC, area under the concentration–time curve; AUC_0–24/last/inf_, AUC from time 0 until the 24 hours/last timepoint; AUC_0–inf_, AUC from time 0 extrapolated to infinity; CL/F, apparent clearance; CLr, renal clearance; C_max_, maximum plasma concentration; f_e_, fraction excreted unchanged in urine; PK, pharmacokinetic; t_½_, terminal elimination half-life; T_max_, time to maximum concentration; %CV, percent coefficient of variation.

^a^ Values are geometric mean (geometric %CV) except where indicated.

^b^ Median (minimum, maximum).

#### **Supplementary Table S6.** Multiple-Dose Etavopivat Plasma PK in Healthy Subjects [Geometric Mean (Geometric %CV) Except Where Indicated]

|  |  | **Etavopivat Dose** | | | |
| --- | --- | --- | --- | --- | --- |
|  |  | 100 mg BID | 200 mg BID | 300 mg BID | 400 mg QD |
| **Parameter^a^** | Day | (n = 9)^b^ | (n = 9)^c^ | (n = 9)^b^ | (n = 9)^d^ |
| AUC_0–tau_, ng∙h/mL | 1 | 470 (36) | 1016 (43) | 1353 (51) | 2465 (31) |
|  | 14 | 563 (36) | 1072 (43) | 2406 (7) | 2962 (21) |
| Ratio AUC_0–tau_ |  | 1.46 (5.2) | 1.27 (47.8) | 1.26 (9.0) | 1.20 (28.4) |
| AUC_0–last_, ng∙h/mL | 1 | 431 (33) | 1058 (43) | 1642 (57) | 2385 (31) |
| AUC_0–inf_, ng∙h/mL | 1 | 530 (35) | 1128 (42) | 1475 (50) | 2671 (30) |
| C_max_, ng/mL | 1 | 129 (58) | 353 (64) | 502 (70) | 760 (49) |
| C_max_, ng/mL | 14 | 141 (31) | 299 (35) | 704 (31) | 668 (32) |
| Ratio C_max_ |  | 1.10 (58.1) | 0.85 (51.1) | 1.40 (49.2) | 0.88 (72.4) |
| T_max_, h^e^ | 1 | 1.00 (1.0, 4.0) | 2.00 (1.0, 4.0) | 2.00 (1.0, 4.0) | 1.00 (1.0, 4.0) |
| t_½_, h | 1 | 5.0 (14.5) | 4.1 (39.5) | 3.7 (36.1) | 10.6 (24.2) |
| CL/F, L/h | 1 | 188.8 (35.3) | 177.4 (41.9) | 203.3 (50.2) | 149.8 (29.7) |
| CL/F_SS_, L/h | 14 | 177.5 (36.1) | 186.6 (43.2) | 124.7 (6.8) | 135.0 (20.7) |

AUC, area under the concentration–time curve; AUC_last_, AUC from time 0 until the last time point; AUC_0–inf_, AUC from time 0 extrapolated to infinity; AUC_0–tau_, AUC from time zero to the end of the dosing period (that is, 0–12 for twice daily and 0–24 for once daily); CL/F, apparent clearance; CLr, renal clearance; C_max_, maximum plasma concentration; PK, pharmacokinetic; t_½_, terminal elimination half-life; T_max_, time to maximum concentration; %CV, percent coefficient of variation.

^a^ Values are geometric mean (geometric %CV) except where indicated.

^b^ n = 6 for day 1 AUC_0-tau_, day 1 AUC_0–inf_, t_½_, and CL/F; n = 3 for day 14 AUC_0-tau_, CL/F_SS_, and Ratio AUC_0–tau_.

^c^ n = 8 for day 1 AUC_0-tau_, day 1 AUC_0–inf_, t_½_, and CL/F; n = 3 for day 14 AUC_0-tau_, CL/F_SS_, and Ratio AUC_0–tau_.

^d^ n = 8 for day 1 AUC_0-tau_, day 1 AUC_0–inf_, t_½_, CL/F, and day 14 Ratio AUC_0–tau_.

^e^ Median (minimum, maximum).

#### **Supplementary Table S7.** Effect of Food on Plasma PK of a 400 mg Single Dose of Etavopivat in Healthy Subjects: Plasma PK parameters

|  | **Etavopivat 400 mg** | |
| --- | --- | --- |
|  | Fasted | Fed |
| Parameter^a^ | (n = 10) | (n = 10) |
| AUC_0–24h_, ng∙h/mL | 1918 (1857) | 2033 (1357) |
| AUC_0–last_, ng∙h/mL | 2031 (1902) | 2234 (1537) |
| AUC_0–inf_, ng∙h/mL | 2054 (1900) | 2262 (1539) |
| C_max_, ng/mL | 596 (617) | 451 (322) |
| T_max_, h^b^ | 1.0 (0.5, 6.0) | 3.0 (0.8, 6.0) |
| t_½_, h | 10.2 (2.6) | 12.0 (3.8) |
| CL/F, L/h | 268.2 (104.1) | 220.6 (86.0) |

AUC, area under the concentration–time curve; AUC_0–24/last_, AUC from time 0 until the 24 h/last time point; AUC_0–inf_, AUC from time 0 extrapolated to infinity; CL/F, apparent clearance; C_max_, maximum plasma concentration; PK, pharmacokinetic; t_½_, terminal elimination half-life; T_max_, time to maximum concentration.

^a^ Values are arithmetic mean (SD) except where indicated.

^b^ Median (minimum, maximum).

#### **Supplementary Table S8.** Effect of Food on Plasma PK of a 400 mg Single Dose of Etavopivat in Healthy Subjects: Statistical Comparison of PK Parameters

|  | **Geometric LS Means** | | **Ratio of Geometric LS Means (Fed/Fasted), %** | **90% CI for Ratio, %** |
| --- | --- | --- | --- | --- |
|  | Fasted | Fed |  |  |
|  | (n = 10) | (n = 10) |  |  |
| AUC_0–24h,_ ng∙h/mL | 1540.88 | 1787.33 | 116.0 | 105.7, 127.3 |
| AUC_0–last,_ ng∙h/mL | 1642.95 | 1953.45 | 118.9 | 110.1, 128.4 |
| AUC_0–inf,_ ng∙h/mL | 1669.61 | 1982.26 | 118.7 | 110.0, 128.1 |
| C_max,_ ng/mL | 447.53 | 366.66 | 81.9 | 60.3, 111.4 |

ANOVA, analysis of variance; AUC, area under the serum concentration–time profile; AUC_0–24h/last_, AUC from time 0 until the 24 hours/ time point of the last quantifiable concentration; AUC_0–inf_, AUC from time 0 extrapolated to infinity; C_max_, maximum concentration; LS, least squares; CI, confidence interval; PK, pharmacokinetic.

An ANOVA model was performed on the log-transformed PK parameter of the 2 treatments (fed versus fasted), including terms for food status, sequence, and period as fixed effects, and subjects-nested-within-sequence as a random effect. A subject must have had a calculable PK parameter in both food statuses to be included in the analysis for that parameter. Geometric LS means are the least squares means from the mixed model presented after back-transformation to the original scale. The 90% confidence intervals are presented after back-transformation to the original scale.

#### **Supplementary Figure S1.** Chemical structure of etavopivat.

#### **Supplementary Figure S2.** Patient flow.


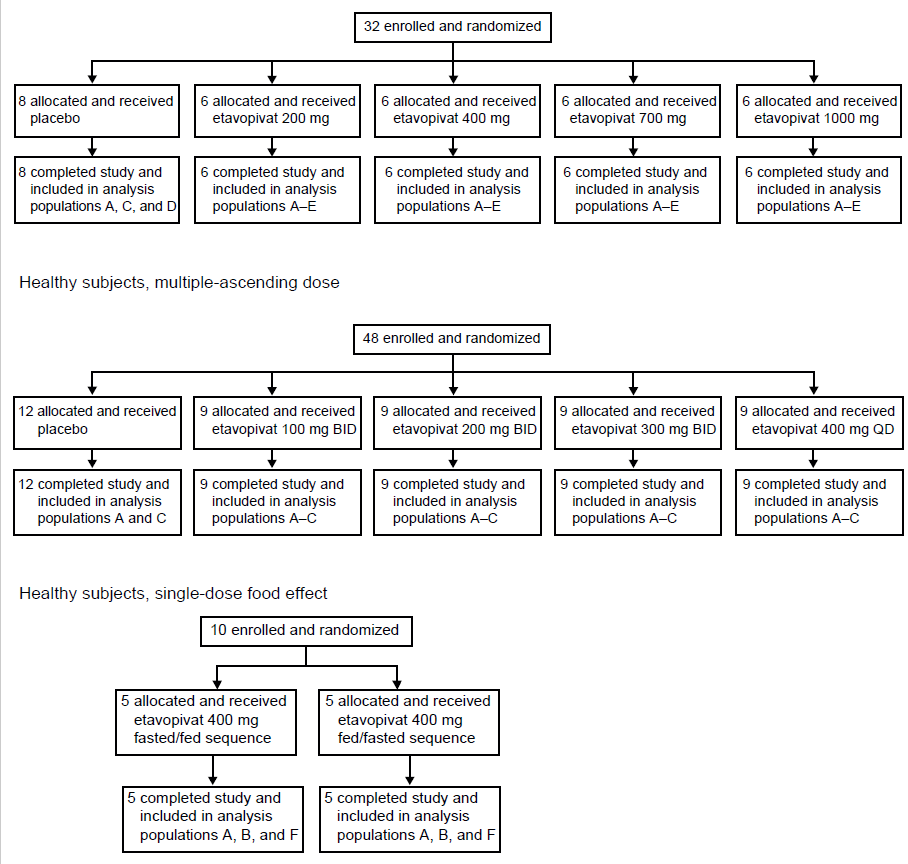


BID, twice daily (every 12 hours); PD, pharmacodynamics; PK, pharmacokinetics; QD, once daily; QTc, corrected QT interval.

Analysis populations: A=Safety; B=PK; C=PD; D=QTc; E=PK/QTc; F=Food effect.

#### **Supplementary Figure S3.** Change from baseline in endogenous (A) estradiol and (B) testosterone in men in the multiple-ascending dose cohorts.

BID, twice daily (every 12 hours); QD, once daily; SD, standard deviation.

Data points are offset for clarity.

#### **Supplementary Figure S4.** Placebo-adjusted change from baseline in QTcF interval (ΔΔQTcF) in healthy subjects receiving a single dose of etavopivat.

Relationship between etavopivat concentration and mean ΔΔQTcF (quantile plot).


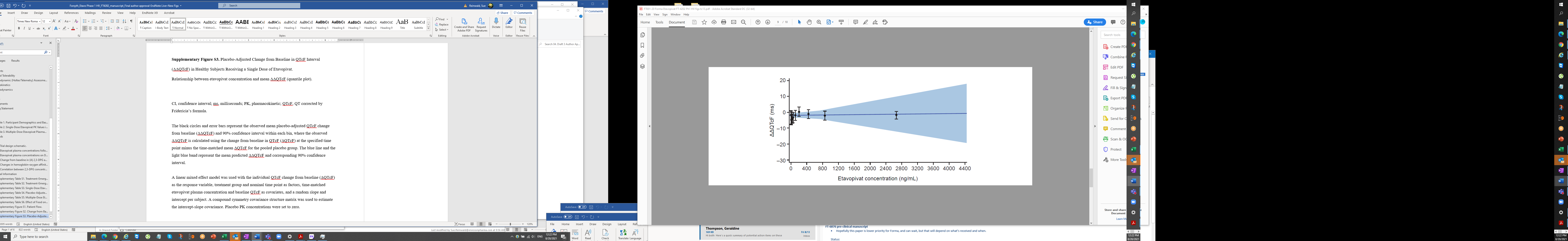


CI, confidence interval; ms, milliseconds; PK, pharmacokinetic; QTcF, QT corrected by Fridericia’s formula.

The black circles and error bars represent the observed mean placebo-adjusted QTcF change from baseline (ΔΔQTcF) and 90% confidence interval within each bin, where the observed ΔΔQTcF is calculated using the change from baseline in QTcF (ΔQTcF) at the specified time point minus the time-matched mean ΔQTcF for the pooled placebo group. The blue line and the light blue band represent the mean predicted ΔΔQTcF and corresponding 90% confidence interval.

A linear mixed-effect model was used with the individual QTcF change from baseline (ΔQTcF) as the response variable, treatment group and nominal time point as factors, time-matched etavopivat plasma concentration and baseline QTcF as covariates, and a random slope and intercept per subject. A compound symmetry covariance structure matrix was used to estimate the intercept-slope covariance. Placebo PK concentrations were set to zero.

#### **Supplementary Figure S5.** Effect of food on plasma PK of a 400 mg single dose of etavopivat in healthy subjects: etavopivat plasma concentrations following a 400-mg single dose with or without food

LLOQ, lower limit of quantitation; SD, standard deviation.

Data points are offset for clarity. Inset shows the interval from 0 to 8 hours on an extended time scale. Concentrations below the LLOQ were set to zero.

#### **Supplementary Figure S6.** Modeling of the predicted PD response (2,3-DPG and ATP) in the RBCs of healthy subjects.


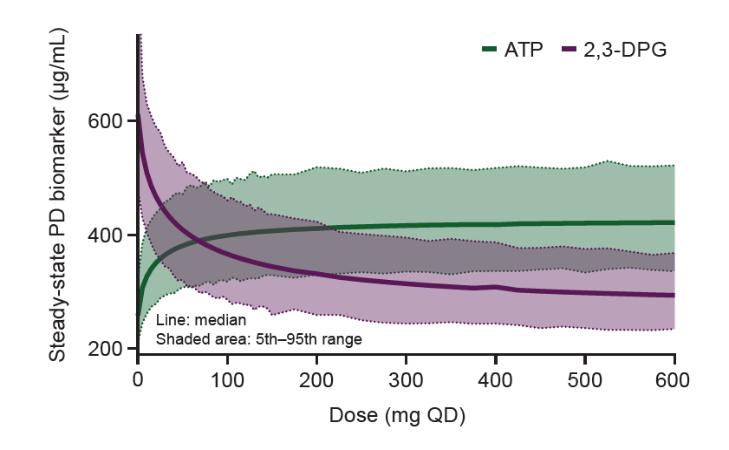


2,3-DPG, 2,3-diphosphoglycerate; ATP, adenosine triphosphate; PD, pharmacodynamic; RBCs, red blood cells.
